# A computational procedure for identifying master regulator candidates: a case study on diabetes progression in Goto-Kakizaki rats

**DOI:** 10.1186/1752-0509-6-S1-S2

**Published:** 2012-07-16

**Authors:** Guanying Piao, Shigeru Saito, Yidan Sun, Zhi-Ping Liu, Yong Wang, Xiao Han, Jiarui Wu, Huarong Zhou, Luonan Chen, Katsuhisa Horimoto

**Affiliations:** 1 School of Life Sciences, University of Science and Technology of China, Hefei 230026, China; 2 Key Laboratory of Systems Biology, SIBS-Novo Nordisk Translational Research Centre for PreDiabetes, Shanghai Institutes for Biological Sciences, Chinese Academy of Sciences, Shanghai 200233, China; 3 Computational Biology Research Center, National Institute of Advanced Industrial Science and Technology, Tokyo 135-0064, Japan; 4 INFOCOM Corporation, Tokyo 150-0001, Japan; 5 Key Laboratory of Human Functional Genomics of Jiangsu Province, Nanjing Medical University, Nanjing 210029, China; 6 National Center for Mathematics and Interdisciplinary Sciences, Academy of Mathematics and Systems Science, Chinese Academy of Sciences, Beijing 100190, China

## Abstract

**Background:**

We have recently identified a number of active regulatory networks involved in diabetes progression in Goto-Kakizaki (GK) rats by network screening. The networks were quite consistent with the previous knowledge of the regulatory relationships between transcription factors (TFs) and their regulated genes. To study the underlying molecular mechanisms directly related to phenotype changes, such as diseases, we also previously developed a computational procedure for identifying transcriptional master regulators (MRs) in conjunction with network screening and network inference, by effectively perturbing the phenotype states.

**Results:**

In this work, we further improved our previous method for identifying MR candidates, by listing them in a more reliable manner, and applied the method to reveal the MR candidates for diabetes progression in GK rats from the active networks. Specifically, the active TF-gene pairs for different time periods in GK rats were first extracted from the networks by network screening. Another set of active TF-gene pairs was selected by network inference, by considering the gene expression signatures for those periods between GK and Wistar-Kyoto (WKY) rats. The TF-gene pairs extracted by the two methods were then further selected, from the viewpoints of the emergence specificity of TF in GK rats and the regulated-gene coverage of TF in the expression signature. Finally, we narrowed all of the genes down to only 5 TFs (Etv4, Fus, Nr2f1, Sp2, and Tcfap2b) as the candidates of MRs, with 54 regulated genes, by merging the selected TF-gene pairs.

**Conclusions:**

The present method has successfully identified biologically plausible MR candidates, including the TFs related to diabetes in previous reports. Although the experimental verifications of the candidates and the present procedure are beyond the scope of this study, we narrowed down the candidates to 5 TFs, which can be used to perform the verification experiments relatively easily. The numerical results showed that our computational method is an efficient way to detect the key molecules responsible for biological phenomena.

## Background

Recent developments in genome-wide computational analyses have successfully identified causal interactions [1], and showed promise in the identification of dysregulated genes in development and tumor progression pathways [2]. For example, a computational analysis procedure was applied to identify the MRs causally linked to the activation of a specific gene set, mesenchymal gene expression signature (MGES), in human malignant glioma [3]. Indeed, 53 TFs were obtained by the ARACNe algorithm and the MGES enrichment test, and among them, the top 6 TFs with the largest fraction of MGES genes were experimentally controlled, as the MR candidates. Finally, 2 of the top 6 TFs, STAT3 and CEBPB, were experimentally verified as MRs of mesenchymal transformation. Unfortunately, the computational method employed in the previous work was unsophisticated and required further improvement. For example, it is unclear why the method selected the top 6 TFs from 53 TFs, rather than 5 or 7 TFs. Although the coverage of the TFs for the MGES genes was carefully considered, there was no rational criterion for the final selection of the MR candidates. Furthermore, ARACNe considers the relationships between three genes for selecting MR candidates. However, there are some well known mathematical techniques that consider multiple relationships and have been applied to infer regulatory networks [4].

We previously reported 39 candidates of active networks for diabetes progression in the Goto-Kakizaki rat (GK), which were identified by network screening, in comparison with the Wistar-Kyoto (WKY) rat [5]. Network screening is a procedure to extract the regulatory networks activated under particular conditions, based on the known regulatory networks [5-7]. The candidates were characterized by the known biological pathways that were consistent with the previous knowledge about diabetes. Unfortunately, the plausibility of the active networks could not be verified experimentally. This was partly because the results were presented in a metaphysical form, and as the biological pathway, instead of the list of concrete target genes. Actually, the active networks were composed of many genes that were not amenable to experimental verification.

To overcome these problems, we recently developed a procedure for identifying MR candidates, by a combination of network screening and network inference [8]. The network screening strongly depends on the previous knowledge of the regulatory networks. To compensate for the limitations of network screening, we introduced a network inference method, which is a version of a path consistency algorithm (PC-A) [9] or a modified PC-A [10,11] that applies PC-A to biological data with high redundancy. The performance of our procedure was tested for MRs in human malignant glioma, using the same data set in ref. [3], and worked well [8]. In total, 22 TFs and 27 TFs were detected by the network screening and the network inference, respectively, and 3 TFs overlapped between them. Interestingly, 2 of the 3 TFs were STAT3 and CEBPB, which were verified experimentally as the master regulators in the previous report [3].

In this paper, we sought to identify the candidates of master regulators for diabetes progression, using the spontaneous diabetic GK rat model. Based on the networks specific to diabetes progression identified in our previous report [5] and the networks inferred by the modified PC-A, we intended to narrow down the candidate molecules responsible for diabetes further, by identifying the master regulators that play a central role in diabetes progression in GK rats. Furthermore, we improved our previous method [8] to narrow down the candidates in a more reliable manner, by considering the coverage of a TF for its regulated genes in a statistical manner, in addition to the specificity of the TF to the target biological phenomena. As expected from the previous case of the computational identification of MRs in a human brain tumor [8] and the present improvements, we identified a limited set of reliable MR candidates, and thus provided information for further experimental design for candidate verification.

## Results

### Overview of our computational procedure

**Figure 1:**
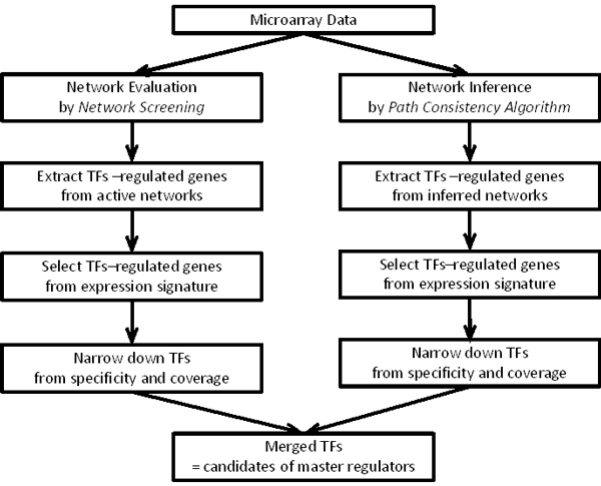
**Workflow of the MR identification procedure**.

In our computational procedure, we identified MR candidates by two approaches, which are schematically shown in Figure 1. One is a knowledge-based approach, which estimates the consistency of the network structures among the known networks with the measured data (named "network screening") [5-7]. The other is a data-driven inference approach, which estimates the conditional independency between the genes by calculating the partial correlation coefficients (named "modified path consistency algorithm") [10,11]. In both cases, we further selected the MR candidates by considering the enrichment of the gene expression signature in the networks. Finally, we refined the candidates by considering the TF specificity and the regulated-gene coverage. The details are described in the Methods.

### MR candidates detected by network screening

In our previous study [5], we used network screening to identify 39 networks for GK and WKY rats in three growth periods: 4w, from 8w to 12w, and from 16w to 20w, among the 1,760 networks in the reference network set. Based on these results, we further selected the MR candidates. From the 39 networks, in total, we extracted 568 binary relationships of TFs and their regulated genes, which were specifically found in the three periods for GK and WKY rats, under the condition that the gene expression shows a difference with a false discovery rate (FDR) of less than 0.05, between the two strains of rats for each period (see the details in the Methods). The numbers of genes specifically found in each period in GK and WKY rats are as follows: 54 genes at the period of 4w in GK; 199 at 8w and 12w in GK; 56 at 16w and 20w in GK; 95 at the period of 4w in WKY; 125 at 8w and 12w in WKY; and 39 at 16w and 20w in WKY. Note that some TF-gene relationships emerged iteratively for different periods in GK and WKY rats.

Among the TF-gene relationships selected above, the TFs were narrowed down in two ways. First, the TF-gene relationships were selected by the specificity, which means that the TFs emerge only in GK, but not in WKY. As a result, we found a total of 21 TFs, as shown in Table 1. Second, the TF-gene relationships were selected by the coverage, which means how many genes the TFs regulate, among the genes in the expression signature. The TFs thus selected were sorted according to the coverage, and then the MR candidates were further selected by a statistical test (see the Methods) for each period in GK and WKY listed in Table 2. As seen in the table, most of the TFs emerged in both GK and WKY, in terms of the coverage selection. We finally found 3 TFs (EGR1, NRF1, and TCFAP2A) among the genes by the initial selection in Table 2.

**Table 1:** TFs identified by network screening in terms of specificity.

**Ar, Bcl6, Brca1, Etv4, Fus, Gli1, Hes1, Hnf1b, Hnrnpk, Klf10, Klf4, Lyl1, Mef2c, Nfia, Nr2f1, Nrl, Pax6, Sp2, Sp4, Tcfap2b, Wt1**

All of the gene names are cited from the Rat Genome Database http://rgd.mcw.edu/ in all of the tables, the figures, and the text.

**Table 2:** TFs identified by network screening in terms of coverage.

4w				8w_12w				16w_20w			
**GK**		**WKY**		**GK**		**WKY**		**GK**		**WKY**	

**TF**	**No. of regulated genes**	**TF**	**No. of regulated genes**	**TF**	**No. of regulated genes**	**TF**	**No. of regulated genes**	**TF**	**No. of regulated genes**	**TF**	**No. of regulated genes**

**SP1**	10	**SP1**	19	**SP1**	39	**SP1**	18	**SP1**	12	**SP1**	5
		**SP3**	8	**SP3**	11	HNF4A	6	**SP3**	3	**FOXO3**	3
		**TP53**	4	**TP53**	11	**FOXO3**	4				
				EGR1	6						
				NRF1	6						
				TCFAP2A	5						

TFs found in both GK and WKY are indicated by bold letters.

### MR candidates inferred by the modified path consistency algorithm

We first inferred six networks of all genes on the microarray for each of the three periods in GK and WKY rats, by the modified path consistency algorithm [10,11], and then the TF-gene relationships were extracted from each network. After the extraction, only the relationships that included the genes with a significant difference between GK and WKY rats were further selected for the 6 sets of relationships.

Using the same procedure as that described in the preceding subsection, the TFs were narrowed down. First, we chose the relationships in terms of the gene-emergence specificity. As a result, 108 TFs were identified as the MR candidates in Table 3. The number of candidates seems to be large, even in comparison with the candidate number, 27 TFs, in the previous case of the brain tumor [3]. While one network was considered to identify the candidates in the previous paper, three networks for the three periods in GK rats were surveyed to select the candidates in the present study. Thus, the number of TFs extracted from one network, 36 TFs on average, is similar to that in the previous study. Second, the TF-gene relationships were selected by the coverage. We chose the TF-gene relationships by a statistical test (see the details in the Methods) for each period in GK and WKY, as shown in Table 4. In contrast to the coverage selection in network screening, only a few TFs emerged in both GK and WKY. Indeed, among the 44 TFs in Table 4, only two TFs (Tbpl1 and Cbfb) emerged in both GK and WKY. Finally, we found 42 TFs as MR candidates.

**Table 3:** TFs identified by network inference in terms of specificity.

Alx1, Arnt, Cebpg, Ddit3, Dlx5, Dmrt2, Dnmt1, Dr1, Ebf1, Elf5, Elk3, Elk4, Erg, Etv4, Etv5, Fev, Fosl1, Foxe1, Foxg1, Foxo3, Foxp4, Gabpb1l, Gfi1, Gtf2a1, Gtf2b, Gtf2e1, Gzf1, Hcfc1, Hey1, Hhex, Hoxb3, Hoxb7, Ilf3, Irx2, Kcnip4, Klf1, Klf15, Klf3, Klf5, Klf7, Ldb2, LOC680117, Mafk, Meis2, Mnat1, Msx1, Msx2, Mybl2, Myc, Myocd, Myod1, Mzf1, Neurod2, Nfix, Nfx1, Nkx6-1, Notch1, Nr1h4, Nr2f1, Nr4a1, Nr5a1, Pax8, Pbx2, Phox2a, Pitx1, Pitx3, Pou2f3, Pou3f1, Ppard, Pparg, Ppargc1a, Rbl1, RGD1566107, Rreb1, Runx1, Shh, Six5, Six6, Skp2, Sox10, Sox11, Sp1, Sp2, Spdef, Srebf1, Ss18l1, Stat5a, Stat5b, Taf2, Tbx18, Tbx2, Tcf12, Tcfap2b, Tead1, Tfdp2, Tfec, Tmf1, Tp53bp1, Twist1, Vdr, Zbtb5, Zfhx3, Zfp191, Zfp238, Zfp423, Zfp444, Zhx1, Zic1

**Table 4:** TFs identified by network inference in terms of coverage.

4w				8w_12w				16w_20w			
GK		WKY		GK		WKY		GK		WKY	
TF	No. of regulated genes	TF	No. of regulated genes	TF	No. of regulated genes	TF	No. of regulated genes	TF	No. of regulated genes	TF	No. of regulated genes
Arntl	31	Max	10	Lhx5	24	Ywhae	18	Fus	10	Foxq1	32
Lhx2	22	Otx2	10	Etv1	23	Pfdn5	13	Smad5	10	Hoxa1	16
Sp2	18	Daxx	9	Ctnnb1	8	Atf1	11	Nfx1	9	Rbl2	16
Gabpa	13	Sim1	9	Rpa3	8	Cdk9	11	Hsf1	8	Zic2	12
Xpa	4	Tcf21	8	Zfp105	8	Hmgb2	11	Tlx3	8	Rorc	8
Foxs1	3	Gata5	7	Foxo3	7	Sfpq	9	Tp53	8	Tcfap4	6
		Tcfap2c	7	Hoxc5	6	Zfp281	9	Foxs1	7	Pttg1	5
		Meis3	5	Litaf	6	Cdk7	8	LOC679869	7	Ncoa3	4
		Rorc	5	Nr2f2	6	Ets2	8	**Cbfb**	6	Ccnh	3
		Snapc1	5	Foxo1	5	Hoxa1	8	Ctcf	6	Hif1a	3
		Zic2	5	Msx1	5	Nfe2l2	8	Glis2	6	Junb	3
		Meis1	4	Myocd	5	Nfil3	8	Irf7	6	Kcnip1	3
		Pou2af1	4	Pbx1	5	Six4	8	Nfkbib	6	Mtf1	3
		Srf	4	**Tbpl1**	5	Cux2	7	Nr1i2	6	Zfp148	3
		Stox2	4	Vdr	5	Mafg	7	Hdac1	5		
		Tcfcp2l1	4	Hltf	4	Nfkbia	7	Rfx5	5		
		Gtf2h2	3	Htt	4	Pgr	7	Tle1	5		
		Zfx	3	LOC680117	4	Ppp1r13b	7	Xpa	5		
				Mbd1	4	**Tbpl1**	7				
				Parp1	4	**Cbfb**	6				
				Rreb1	4	Ezh2	6				
				Smarcc1	4	Hbp1	6				
						Junb	6				
						Taf13	6				
						Tef	6				

TFs found in both GK and WKY are indicated by bold letters.

### MR selection by comparison of the TF sets detected by the two methods

We obtained the final MR candidates by selecting the overlapped TFs detected by the two methods in terms of two criteria (Tables 1, 2, 3, 4), as shown in Table 5. Indeed, 21 TFs detected by network screening in terms of specificity overlapped with only 4 TFs (Etv4, Nr2f1, Sp2, and Tcfap2b) and 2 TFs (Fus and Sp2) by the modified path consistency algorithm by two criteria, respectively. In contrast, 3 TFs detected by network screening in terms of coverage showed no overlapped TFs by the path consistency algorithm by two criteria. This difference might reflect the restriction of the known TF-gene relationships in network screening.

**Table 5:** Summary of TFs identified by the two methods, in terms of specificity and coverage.

		path consistency algorithm	
		specificity (108)	coverage (42)
network screening	specificity (21)	4	2
	coverage (3)	0	0

As a result, we merged the MR candidates identified by the two methods, and 5 TFs were finally identified as the candidates of MRs for diabetes progression in GK rats. Note that Sp2 emerged in both the 4 TFs and 2 TFs. The 5 final MR candidates with their regulated genes, in total 54 genes, are listed in Table 6.

**Table 6:** Candidates of MRs and their regulated genes for diabetes progression in GK rat.

TF	Regulated genes						No. of genes	
Etv4	Mcm10	**Erbb2**	**Mmp7**	**Nid1**	Plau	Ptgs2	6	
Fus	Mcpt8l2	Mcpt9	**Paics**	**Ppat**	Ugt1a1	Ugt1a2	12	54
	Ugt1a3	Ugt1a5	Ugt1a6	Ugt1a7c	Ugt1a8	Ugt1a9		
Nr2f1	Alox5	**Cpt1b**	Cyp11b2	**Tf**	Ugt1a3	Ugt1a5	6	
Sp2	**Capns1**	**Irs2**	LOC685183	LOC685226	LOC685291	LOC685759	24	
	LOC688519	LOC688603	LOC689083	LOC689312	LOC689338	LOC689690		
	LOC689999	LOC690179	LOC690328	LOC690379	LOC690577	LOC691712		
	LOC691735	LOC691754	Papss2	Vom2r45	Vom2r46	Vom2r47		
Tcfap2b	Aqp1	Egfr	Krt14	Ptgds	Sod2	Tgm1	6	

The genes in bold characters are included in known TF-gene relationships detected by network screening.

## Discussion

In this study, we have identified the candidates of master regulators based on our previous study [5], by using an improved method for their identification [8]. The MR candidates were extracted from the active networks of many genes characterized by biological pathways, as the feasible gene candidates for experimental verification. From the methodological aspect, the method was improved by considering the coverage of TFs in a statistical manner, in addition to the specificity that was considered in the previous method. Although the experiments are beyond the scope of the present study, we consider experimental verification studies of the present candidates as our future research topic. Our study clearly illustrated a rational way to narrow down the genes of MR candidates, and is fundamentally different from metaphysical presentations, such as biological pathways or large network forms.

**Figure 2:**
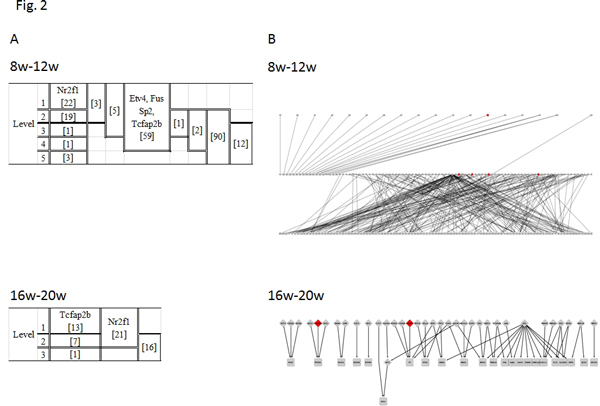
**Hierarchical structures of networks for 8w-12w and 16w-20w by two previous methods**. The 5 TFs are indicated at the levels in hierarchical structures obtained by the vertex-sort algorithm (A) [12] and those by the BFS method (B) [13], and the numbers of TFs in each level are indicated in parentheses in (A), and by red circles in (B). In (B), the TFs and the regulated genes are indicated by diamonds and rectangles, respectively.

Our study intended to identify the MR candidates, which are those genes with large impacts on phenotype changes, in a biological sense [3]. Here, we logically identified MR candidates by the specificity of the TF appearance and the coverage of the regulated genes to the gene expression signature in the networks of GK and WKY rats. Apart from a biological sense, we further investigated the meaning of "master" from the viewpoint of the network structure. To do this, we revealed the hierarchical structures of the 8w-12w and 16w-20w networks by network screening, using a vertex sort algorithm [12], and allocated the present 5 TFs into the hierarchical structures (Figure 2A). As seen in the figures, all 5 TFs were allocated into the highest level. Indeed, Nr2f1 in the 8w-12w network and Tcfap2b in the 16w-20w network were definitely allocated into the highest level of the hierarchical structures. In addition, the remaining TFs were allocated into the levels including the highest and middle levels, but not into the lowest level. Furthermore, we investigated the hierarchical structure by another method, the BFS-level algorithm [13]. As shown in Figure 2B, the positions of the MR candidates are similar to those in Figure 2A. Indeed, previous hierarchical analyses of the regulatory networks by the BSF method in *Escherichia coli *and *Saccharomyces cerevisiae *suggested that the MRs were in the middle of the hierarchy [13]. In general, the vertex sort algorithm reports a linear ordering of nodes that contains all feasible solutions, while the BSF-level algorithm reports just a single solution, as shown in Figures 2A and 2B. Subsequently, unlike the BFS-level algorithm, the ordering in the vertex sort algorithm permits nodes to span an entire interval of possible positions with any feasible ordering. Despite this difference in the computational algorithms, the 5 TFs showed the common property as MRs. At any rate, although the verification experiments remain to be performed for the justification of the MRs in a biological sense, the 5 TFs may be regarded as the plausible MR candidates from the viewpoint of network structure.

A preliminary survey revealed that all 5 of the TFs have no reported causal relationship to diabetes. The 5 TFs are sequence-specific DNA-binding proteins, and they function as both transcriptional activators and repressors of large numbers of genes that are closely related to the cell cycle and tumorigenesis. Notably, the relationships of ETS translocation variant 4 (Etv4) and transcription factor AP-2 beta (Tcfap2b) to adipogenesis, which is strongly related to diabetes, have been reported, together with their association with the other pathways [14,15]. Nuclear Receptor subfamily 2, group F, member 1 (NR2F1) is a member of the steroid hormone receptor family, and has been shown to interact with estrogen receptor alpha (ESR1) [16]. There is a gender difference in the incidence of type 2 diabetes, which is largely due to the role of the sex hormone estrogen. The Sp family proteins, containing the conserved DNA-binding domain, are localized primarily within subnuclear foci associated with the nuclear matrix. Recent unpublished data from our lab have shown that another Sp family member, Sp1, has a major impact on the insulin signaling pathway. The Sp2 transcription factor interacts with E2F1, which mediates both cell proliferation and p53-dependent/independent apoptosis [17]. The recently discovered close relationships between diabetes and tumors in terms of these TFs are quite likely to play a crucial role in the control of diabetes. RNA-binding protein (FUS) is able to bind DNA, RNA and protein [18]. The interactions between the FUS recognition sites and Tcfap2, GCF, and Sp1 were identified recently. Thus, although direct evidence was not found in the previous knowledge, the 5 TFs are expected to be MR candidates, in consideration of the circumstantial evidence of their relationships to diseases, the hierarchical analysis of the 5 TFs, and the successful discovery of new MRs in brain tumor, by the previous version of the procedure. Actually, our current information in terms of important diabetes-related genes includes mostly functional proteins, located at the lowest level of our hierarchical structure, while the MR is deeply hidden and therefore must be revealed by systems biology methods. Thus, in addition to analyses of their regulated genes, some experimental verification of the MR candidates may be desirable to further examine their plausibility as MR candidates for diabetes progression.

## Conclusions

In this work, using our new method, we identified the MR candidates for diabetes progression, 5 TFs and their regulated genes, in GK rats. This number of candidates is very small, and thus the results can be used as a basis for biological experiments for verification. Furthermore, the recent availability of the next-gen sequencer may provide another way to confirm the effectiveness of our method, and to test its performance further with other datasets. Indeed, RNA-seq and ChIP-seq are useful for more accurate measurements of gene expression, and yield detailed information about the regulated genes. Thus, the combined use of the two approaches may compensate for the pitfalls inherent in each method, and will provide important clues about the transcriptional networks that regulate transitions into physiological or pathological cellular states.

## Methods

### Network screening

The candidates of the active regulatory networks were detected by network screening [5-7]. Here, we briefly summarize the network screening in the present study, as follows.

First, the regulatory network sets were generated in the same manner as in the previous study [5], as follows. The mouse binary relationships compiled in the TRANSFAC database [19] were used. Based on the correspondence between the mouse and rat gene ids, 3,015 binary relationships of 1,507 genes between 503 TFs and 1,123 regulated genes were achieved. Based on those binary relationships, transcriptional networks were constructed according to the functional gene sets previously defined in the Molecular Signatures Database (MSigDB) [20]. In each gene set, the regulated genes in the binary relationships were searched, and if at least one gene was found in the gene set, then the corresponding binary relationships were regarded as a regulatory network characterized by the gene set. In present study, the reference network comprised 1,760 regulatory networks characterized by biological functions that are composed of 1,195 genes. The numbers of TFs and regulated genes were 335 and 860, respectively.

Then, we calculated the graph consistency probability (GCP) [6], which expressed the consistency of a given network structure with the monitored expression data of the constituent genes in this study. The consistency of a directed acyclic graph (DAG), *G*(*V_i_*, *E_j_*), where *V_i _*is a vertex (*i *= 1, 2, ..., *n*_v_) and *E_j _*is an edge (*j *= 1, 2, ..., *n*_e_) in the graph, and the joint density function *f *(*X_i_*), corresponding to *V_i _*for the graph *G *with the measured data, is quantitatively expressed by the logarithm of the likelihood based on the Gaussian graphical model (*GN*: Gaussian Network), i.e.,

(1) $$\begin{array}{ccc} l\left(G_{0}\right) & =\ln\overset{n_{v}}{\underset{i=1}{\prod }}f\left(X_{i}|pa\left\{X_{i}\right\}\right) & \\ \; & =-\frac{1}{2}\overset{n_{v}}{\underset{i=1}{\sum }}\overset{n_{i}}{\underset{j=1}{\sum }}\left\{\frac{1}{\sigma_{i}^{2}}\overset{m}{\underset{k=1}{\sum }}{\left(x_{ik}-\overset{n_{i}}{\underset{j=1}{\sum }}\beta_{ij}x_{kj}\right)}^{2}+\ln\left(2\pi \sigma_{i}^{2}\right)\right\}, \end{array}$$

where *pa*{*X_i_*} is the set of variables corresponding to the parents of *V_i _*in the graph, *x_ik _*is the measured value of *X_i_*, at the *k*-th point, and *n_i _*is the number of variables corresponding to the parents of *V_i_*. Since the likelihood depends on the graph size, we designed a simple procedure to transform the likelihood to the probability for the expression of the graph consistency with the data [6]. First, we generated *N_r _*networks under the condition that the networks shared the same numbers of nodes and edges as those of the given networks. Then we defined GCP, as follows,

(2) $$GCP=\frac{N_{s}}{N_{r}},$$

where *N_s _*is the number of networks with larger log-likelihoods than the log-likelihood of the tested network. In the present study, *N_r _*was set to 2,000, and the *GCP *significance of the given network was set at 0.05.

### Path consistency algorithm

**Figure 3:**
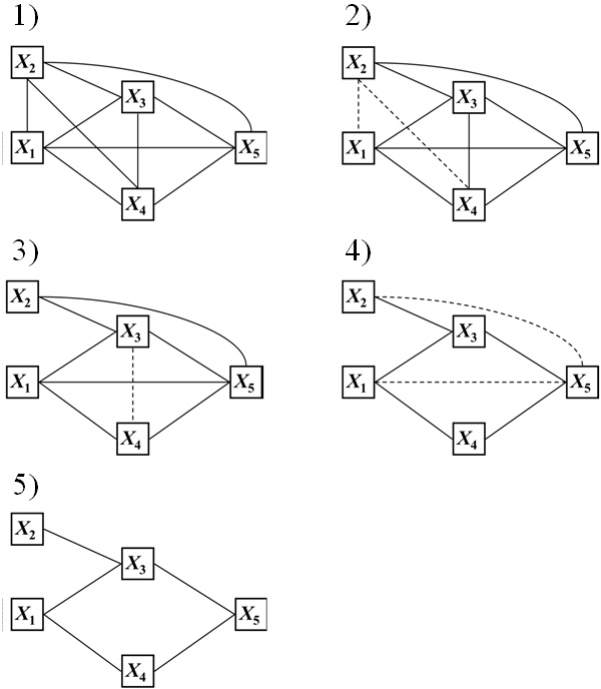
**Example of the path consistency algorithm**.

The path consistency (PC) algorithm [9] is an algorithm to infer a causal graph composed of two parts: the undirected graph inference by a partial correlation coefficient and the following directed graph construction by the orientation rule. The present method partially exploits the first part of the PC algorithm for the inference of the network structures. A simple example of the PC algorithm is illustrated in Figure 3.

We assume that five variables, *X*_1_, *X*_2_, *X*_3_, *X*_4_, *X*_5_, have the following five relationships: i) *X*_1_∐*X*_2_,

ii) *X*_2_∐ (*X*_1_, *X*_4_),

iii) *X*_3_∐*X*_4_|(*X*_1_, *X*_2_),

iv) *X*_4_∐ (*X*_2_, *X*_3_)|*X*_1_, and

v) *X*_5_∐ (*X*_1_, *X*_2_)|(*X*_3_, *X*_4_),

where the symbol, ∐, in the above relationships, means the independence between variables. The PC algorithm reconstructs the above relationships as follows.

1) Prepare a complete graph, *C*, between the five variables.

2) Test the correlation between two variables by calculating the zeroth-order of the partial correlation coefficient (Pearson's correlation coefficient). From the test, two variable pairs, (*X*_1_, *X*_2_) and (*X*_2_, *X*_4_), are excluded (dashed lines in Figure 2), due to the relationships, i) and ii).

3) Test the correlation between three variables by calculating the first-order of the partial correlation coefficient of the variable pairs, given one variable. Then, one variable pair, (*X*_3_, *X*_4_), is further excluded from the updated graph by 2), due to iii) and iv).

4) Test the correlation between four variables by calculating the second-order of the partial correlation coefficient of the variable pairs, given two variables. Then, two variable pairs, (*X*_1_, *X*_5_) and (*X*_2_, *X*_5_), are excluded, due to iv).

5) We could not find any edges adjacent to the three edges in the updated *C*. Thus, the algorithm naturally stops. As seen in the final graph, the five relationships emerged completely.

In general, the (*m*-2)-th order of the partial correlation coefficient is calculated between two variables, given (*m*-2) variables; i.e., *r_ij, rest_*, between *X_i _*and *X_j_*, given the '*rest*' of the variables, {*X_k_*} for *k *= 1, 2, ..., *m*, and *k*≠*i*, *j*, and after calculating the (*m*-2)-th order of the partial correlation coefficient, the algorithm naturally stops. However, the algorithm does not usually request the (*m*-2)-th order of the correlation coefficient for the natural stop. This is because after excluding the variables, the adjacent variables are often not found, even in the calculation of the lower orders of partial correlation coefficients.

### Modification of the path consistency algorithm for microarray data analysis

In the actual expression profile data, many genes frequently show profiles with similar patterns. This makes the numerical calculation of correlation coefficients difficult, due to the multi-colinearity between the variables. The original PC algorithm accidentally stops, if only one correlation between a pair of variables shows a violation of the numerical calculation. However, in a biological sense, the gene pairs that cause the accidental stop can be interpreted as a case of their high association with each other, in terms of gene expression. Thus, we modified the original PC algorithm to prevent it from accidentally stopping with the highly associated gene pairs, as follows [10,11]. If the calculation of any order of the partial correlation coefficient between the variables is violated, then the corresponding pair of variables is regarded as being dependent. For example, if the first-order correlation coefficient, *r_ij, k_*, cannot be calculated numerically, due to the multi-colinearity between *X_i _*and *X_j_*, then the edge *X_i_*-*X_j _*is kept without the statistical test. The other parts remain unchanged in the modified algorithm. Note that the above modification ensures that the algorithm will naturally stop for the data including a high correlation.

As seen in the original algorithm, the output is not unique, depending on the calculation order of pairs [9]. A permutation test for the calculation order is a convenient way to partly resolve this issue. In this study, the estimation without permutation was empirically adopted as the first approximation, based on the successful estimations of the relationships in our previous studies [10,11]. In addition, one of the most remarkable features of the PC algorithm is that the algorithm removes the pseudo-correlations between the variables (genes) by considering the higher-order partial correlations. If we have the measurement data for a complex network, then we frequently face the more serious issue of the pseudo-correlation, rather than the correlation level. The merit of the PC algorithm may be its ability to identify real relationships between TFs and their regulated genes.

### Definition of MR candidates by network screening and network inference

We first referred to two sets of networks obtained by the network screening [5-7] and the network inference [10,11]. From each network set, the binary relationships between the TFs and their regulated genes were extracted, only if the regulated genes were included in the expression signature, which is the ensemble of genes with significant differences in gene expression, as statistically estimated by the false discovery rate (FDR) test for multiple comparisons (FDR < 0.05) [21]. In the extraction of TFs and their regulated genes, the TF was also cited from the TRANSFAC database [19], but the expression degree of the TF was not considered, due to the small expression changes even under different conditions. Only the regulated genes that were estimated to directly bind TFs were extracted. The numbers of genes in the three gene expression signatures of the three periods (period of 4w, period of 8w and 12w, and period of 16w and 20w) were 1,582, 2,719, and 2,777, respectively.

Then, we defined the MR candidates from the binary relationships by two criteria. One was the specificity of the TF, which was the same criterion as in the previous method [8], and the other was the coverage of the TF, which was newly introduced in the present MR candidate identification. Here, the specificity simply means that the TF emerged only in the GK networks, but not in the WKY networks. To select the TFs in terms of the specificity, we selected the TFs that emerged in the three periods in GK, but not in WKY, as the MR candidates. Note that in the selection of the TFs, we only selected those that were estimated to regulate the genes including the expression signature, to consider the enrichment of the regulated genes in the signature. The coverage means how many genes each TF regulates. To select the TFs in terms of the coverage, we first counted the genes regulated by each TF for each period in GK and WKY, and then also considered the enrichment of their regulated genes in the expression signature, by sorting the numbers of regulated genes for each case. To consider the coverage in a rational way, we used the Smirnov-Grubbs outlier test [22] for the numbers of regulated genes, by setting a threshold (p < 0.05). Thus, the TFs with the larger number of regulated genes that fulfilled the threshold are selected in a statistical manner. Finally, the two sets of MR candidates that were selected in terms of the specificity and the coverage were compared, to define the final MR candidates.

### Data analyzed in this study

We analyzed the gene expression data measured in GK and WKY rats [23], which were cited from the National Center for Biotechnology Information (NCBI) Gene Expression Omnibus (GEO; http://www.ncbi.nlm.nih.gov/projects/geo/) database (GSE 13271). The data were composed of 31,099 probes that were measured by using Affymetrix Microarray Suite 5.0 (Affymetrix), and were further reduced into 14,506 genes, for 5 samples of male spontaneously diabetic GK rats and WKY controls at each of 5 time points (4, 8, 12, 16, and 20 weeks of age). In this analysis, the 5 periods were classified into three periods: period of 4w, period of 8w and 12w, and period of 16w and 20w.

## Competing interests

The authors declare that they have no competing interests.

## Authors' contributions

HZ, LC and KH conceived the research. SS, GP, YS and ZPL performed the study. JW, YW and XH provided valuable suggestions and improvements. HZ, LC and KH supervised the project. HZ, ZPL, SS and KH drafted a version of the manuscript. All authors wrote and approved of the manuscript.
